# 

**Published:** 1908-11-07

**Authors:** 


### NOTES, QUESTIONS, AND COMMENTS.

The editor of the Resident Medical Officers' Department of The Hospital invites contributions to this column both from resident medical officers and from other members of the profession. Short articles, notes, queries, or suggestions bearing upon this branch of medical practice will always receive consideration, and those that are suitable will be published in due course. Correspondence, short and to the point, is particularly invited.

Items of news from the resident officers' quarters of the principal hospitals, infirmaries, and asylums throughout the kingdom are invited, and, when of sufficient general interest, will be published. The editor will also be pleased to receive and reply to confidential communications from resident medical officers and to consider any suggestions that may be made for the improvement of this department of The Hospital.

### BOOKS RECEIVED.

J. and A. Churchill.

"Dictionary of Medical Treatment." By Arthur Latham, M.A., M.D.

Kegan, Paul, Trench, Trubner and Co.

"A Text Book of Diseases of the Ear." By Macleod Yearsley, F.R.C.S.

P. S. King and Son.

"The Lament of the Sweated." By James Samuelson.

Longmans, Green and Co.

"Quain's Anatomy." Vol. III. Neurology. By E. A. Schäfer, LL.D., and J. Symington, M.D.

THE

### GRADUATES' MEDICAL SCHOOLS.

DIARY FOR WEEK NOV. 9 TO NOV. 14.

### THE WEST-END HOSPITAL FOR NERVOUS DISEASES, Welbeck Street, W.

**At 5 p.m.**

**Nov. 12**, Mr. Laming Evans, **The Surgical Treatment of Spastic Paralyses.**

### ST. JOHN'S HOSPITAL FOR DISEASES OF THE SKIN, Leicester Square, W.C.

**At 6 p.m.**

**Nov. 12**, Chesterfield Lecture, **Pityriasis Rubra Pilaris and Lichen.**

### THE THROAT HOSPITAL, Golden Square, W.

**At 5.30 p.m.**

**Nov. 9**, Mr. Parker, **Nasal Polypus.**

**Nov. 12**, Mr. Rose, **Inflammation of the Accessory Sinuses of the Nose.**

### THE POST-GRADUATE COLLEGE, West London Hospital, Hammersmith, W.

**At 10 a.m.**

**Nov. 9 and 12**, Mr. Etherington-Smith, **Demonstration of Surgical Cases.**

**At 12 noon.**

**Nov. 9**, Dr. Low, **Pathological Demonstration.**

**At 12.15 p.m.**

**Nov. 13**, Dr. Pritchard, **Practical Medicine.**

**At 5 p.m.**

**Nov. 9**, Dr. Davis, **Diseases of the Middle Ear.**

**Nov. 10**, Dr. Moullin, **Gynæcology, II.**

**Nov. 11**, Dr. Pritchard, **On the Vaccination Treatment of Infective Diseases.**

**Nov. 12** Mr. Keetley, **Clinical, I.**

**Nov. 13**, Dr. Seymour Taylor, **Rheumatoid Arthritis.**

### LONDON SCHOOL OF CLINICAL MEDICINE, Seamen's Hospital, Greenwich, S.E.

**At 2.15 p.m.**

**Nov. 11**, Dr. F. Taylor, **The Treatment of Heart Disease.**

**At 3.15 p.m.**

**Nov. 13**, Mr. McGavin, **Tuberculosis of the Male Genital Tract.**

### MOUNT VERNON HOSPITAL, Hampstead.

**At 5 p.m.**

**Nov. 12**, Dr. A. H. Pirie, **X-Rays in the Diagnosis of Diseases of the Chest.**

### NORTH-EAST LONDON POST-GRADUATE COLLEGE, Prince of Wales's General Hospital, Tottenham, N.

**At 4.30 p.m.**

**Nov. 12**, Dr. Whipham, **Acute Lymphocythæmia in Children.**

### MEDICAL GRADUATES' COLLEGE AND POLYCLINIC, 22 Chenies Street, W.C.

**At 5.15 p.m.**

**Nov. 9**, Mr. Tubby, **Surgical Diseases of Children.**

**Nov. 10**, Dr. Herman, **Neurasthenia in Women.**

**Nov. 11**, Mr. Mayo Robson, **Intervisceral Gall-bladder Fistulæ.**

**Nov. 12**, Dr. Mercier, **Some Interesting Mental Conditions.**

### THE HOSPITAL FOR SICK CHILDREN, Great Ormond Street, W.C.

**At 4 p.m.**

**Nov. 12**, Dr. Hutchison, **Some Common Symptoms of Disease in Children and their Significance.**

### ROYAL COLLEGE OF PHYSICIANS, Pall Mall East.

**At 5 p.m.** FitzPatrick Lectures.

**Nov. 10**, Dr. Leonard Guthrie, **The History of Neurology.**

**At 5 p.m.** Horace Dobell Lectures.

**Nov. 12**, Mr. Leonard Dudgeon, **The Latent Persistence and the Reactivation of Pathogenic Bacteria in the Body.**

DETAILS have lately been published of the Liebig Company's large cattle-farms in the Argentine, Uruguay, and Paraguay. The makers of Lemco, Oxo, and Nursing Oxo possess their own farms of one million and a quarter acres, and their own cattle, numbering 250,000. Every animal is subjected to rigorous tests, both during life and post-mortem, and scientific methods are made use of throughout the process of manufacture. *Lemco* is a pure extract of beef without flavouring, preservative, or addition of any kind. It is free from fat, and will keep good indefinitely in any climate. *Oxo* is a pure beef beverage obtained by amalgamation of pure beef-extract with the albumin and fibrin of selected lean beef, and is seasoned ready for use. During the past twelve months it has been adopted by 76 hospitals, infirmaries, etc. *Nursing Oxo* is a compound of meat-peptone, meat-extract, and meat-fibrin.

